# *Pantoea* species causing early onset neonatal sepsis: a case report

**DOI:** 10.1186/s13256-015-0670-0

**Published:** 2015-09-04

**Authors:** Shreekant Tiwari, Siba Shankar Beriha

**Affiliations:** 1 Department of Microbiology, Hi-Tech Medical College & Hospital, Bhubaneswar, Odisha India; 2 SVBP Post Graduate Institute of Paediatrics, Shishu Bhawan, Cuttack, Odisha India; 3 Kathagola Sahi, Mangalabag, Cuttack, Odisha PIN-753001 India

## Abstract

**Introduction:**

*Pantoea agglomerans* is a plant pathogen which very rarely causes an opportunistic infection. Human beings are usually infected by thorn prick injuries or by contaminated parenteral fluids. *Pantoea agglomerans* has been reported as a cause of neonatal sepsis very rarely and to the best of our knowledge this is the first reported case from India.

**Case presentation:**

A 4-day-old Asian baby boy from the rural area of Odisha, India, was admitted to our neonatal intensive care unit when he presented with fever, tachypnea and chest retraction. *Pantoea* species were isolated from his blood culture.

**Conclusions:**

He was treated successfully with meropenem administered intravenously and other supportive measures. Early detection and proper management may cause a favorable outcome.

## Introduction

*Pantoea agglomerans*, formerly known as *Enterobacter agglomerans*, is a member of family *Enterobacteriaceae*. It is primarily an environmental and agricultural organism that inhabits plants, soil and water. It is an opportunistic pathogen and very rarely causes disease in healthy individuals [1]. The most common infections caused by *Pantoea agglomerans* are septic arthritis or synovitis, cholelithiasis, occupational respiratory infections and skin allergy, peritonitis and blood stream infection in an elderly person [2]. It is an unusual pathogen in the etiology of neonatal sepsis. Here we report a blood stream infection caused by *Pantoea* species (spp.) in a term baby which was delivered vaginally and this is the first documented case from India.

## Case presentation

A 4-day-old Asian baby boy was delivered vaginally at term in our hospital; he was referred to our neonatal intensive care unit (NICU) 48 hours after delivery when he presented with fever, tachypnea and chest retraction. His birth weight was 2.9kg and he cried immediately after delivery. His mother was 19-years old and she came from a rural area. Her antenatal history revealed that she had undergone regular checkups at our hospital; she had no bad obstetric history during the whole pregnancy period. On examination it was found that her human immunodeficiency virus (HIV), hepatitis B virus (HBV) and hepatitis C virus (HCV) status was negative by enzyme-linked immunosorbent assay (ELISA) method. No vaginal lesions were identified during pregnancy and there was no history of birth trauma. She gave a history of premature rupture of membrane (PROM) approximately 8 to 10 hours before delivery. She noted a foul smelling discharge after rupture of membrane.

The baby boy had respiratory distress with some pneumonic changes seen on chest X-ray. A blood sample was collected with complete aseptic precaution into aerobic and anaerobic blood culture bottle (BacT/ALERT® 3D; bioMérieux, Marcy-l’Etoile, France). He was then empirically treated with a combination of amikacin and vancomycin. Aliquots of broth were subcultured on 5% sheep blood agar and MacConkey agar. On blood agar plate colonies were pin-point and had a smooth surface, whereas on MacConkey agar there was a lactose-fermenting colony with similar morphology. A routine biochemical test showed that the colony belonged to the family *Enterobacteriaceae* and further identification was performed by Vitek 2 using GN25 card (bioMérieux, Marcy-l’Etoile, France); it was identified as *Pantoea* spp. with 97% probability. Isolates were sensitive to imipenem, meropenem, amikacin, cefoperazone-sulbactam, ceftriaxone and netilmicin, and resistant to gentamicin, cefotaxime and ceftazidime-clavulanic acid. The anaerobic culture bottle showed no growth after 7 days of incubation. A high vaginal swab of the mother was also sent for culture and sensitivity and it also showed growth of *Pantoea* spp. Other laboratory parameters were: hemoglobin (Hb) 10gm %, red blood cell (RBC) 4.49/cmm^3^, total leukocyte count (TLC) 41,000, neutrophil 80%, lymphocyte 16%, monocyte 4%, C-reactive protein (CRP) 22.5mg/L, urea 73.5mg/dl, serum creatinine 0.45mg/dl, sodium (Na^+^) 141mmol/L and potassium (K^+^) 4.8mmol/L. After collection of the blood sample the baby boy was treated empirically with vancomycin and amikacin. His therapy was changed to meropenem and he was successfully treated with 1 gram twice daily for 14 days after getting the sensitivity report.

## Discussion

*Pantoea* spp. is an opportunistic pathogen and rarely causes disease in healthy individuals. Infections caused by *Pantoea* spp. have been reported in samples obtained from cotton swabs, intra-arterial devices as well as plants and plant material [3]. Cotton swabs are continuously used by nurses and physicians in hospital and can be contaminated in many ways. *Pantoea* spp. is often associated with outbreaks due to contaminated intravenous solutions and stored blood products. *Pantoea* spp. when involved in a systemic infection has a predilection for the lungs.

The common pathogens causing early onset neonatal sepsis are *Klebsiella pneumoniae, Serratia marcescens,* group B streptococcus*, Escherichia coli,* coagulase-negative staphylococci and *Pseudomonas* spp. [4, 5]. *Pantoea* spp. is a very rare pathogen causing early onset neonatal sepsis. To the best of our knowledge this is the first case report of *Pantoea* spp. causing early onset neonatal sepsis from India in an otherwise term baby exposed to PROM. All the previous case reports had been associated with significant prematurity and comorbidity but a similar case report in a near-term baby was reported by Lalas and Erichsen [6] (see Table 1).

**Table 1:** Summary of cases of *Pantoea agglomerans* causing neonatal sepsis

Study group and Reference number	GA (weeks)	BW (gram)	Gender	AD (days)	Comorbidity	Symptoms	IC	CF	Outcome
This study	FT	2900	M	4	PROM, RDS	RD	No	No	Survived
Lalas and Erichsen [6]	35	1990	F	2	PROM	RD	No	No	Good
Bergman *et al*. [10]	29	1795	UK	5	RDS, PROM	RD, shock, DIC	Yes	No	Death
Bergman *et al*. [10]	28	60	UK	20	IUGR, RDS	RD, shock, DIC	Yes	No	Death
Bergman *et al*. [10]	40	3810	UK	12	CHD, PDA	RD, shock, DIC	Yes	No	Death
Habsah *et al.* [11]	26	950	UK	11	RDS	RD, shock, DIC	Yes	Yes	Death
Habsah *et al.* [11]	40	3300	UK	4	Asphyxia	RD, shock, DIC	Yes	Yes	Death
Habsah *et al.* [11]	32	1500	UK	3	RDS	RD, shock, DIC	Yes	Yes	Survived
Habsah *et al.* [11]	40	3200	UK	5	VACTERL	RD, shock, DIC	Yes	Yes	Death
Habsah *et al.* [11]	36	1670	UK	5	IUGR	RD, shock, DIC	Yes	Yes	Death
Habsah *et al.* [11]	36	2000	UK	4	Asphyxia	RD, shock, DIC	Yes	Yes	Death
Habsah *et al.* [11]	26	1200	UK	5	RDS	RD, shock, DIC	Yes	Yes	Death
Cruz *et al.* [9]	FT	UK	F	24	Cardiomyopathy	UK	Yes	No	Death
Cruz *et al.* [9]	FT	UK	M	16	Coarctation	UK	Yes	No	Survived
Aly *et al.* [12]	30	1500	M	13	RDS	RD, Shock	Yes	No	Survived
Aly *et al.* [12]	29	1030	M	12	RDS	RD, Shock	Yes	No	Survived
Aly *et al.* [12]	28	815	F	17	Pre-NEC	RD, Shock	Yes	No	Survived
Aly *et al.* [12]	26	855	M	8	RDS, PDA	RD, Shock	Yes	No	Survived
Aly *et al.* [12]	27	1020	F	11	RDS, PDA	RD, Shock	Yes	No	Survived

*AD* age at diagnosis, *BW* birth weight, *CF* contaminated fluids, *CHD* congestive heart disease, *DIC* disseminated intravascular coagulation, *F* female, *FT* full term, *GA* gestational age, *IC* indwelling catheters, *IUGR* intrauterine growth retardation, *M* male, *NEC* necrotizing enterocolitis, *PDA* patent ductus arteriosus, *PROM* prolonged rupture of membrane, *RD* respiratory distress, *RDS* respiratory distress syndrome, *UK* unknown, *VACTERL* vertebral anomalies, anal atresia, cardiovascular anomalies, tracheoesophageal fistula, esophageal atresia, renal anomalies, limb anomalies

Infections caused by *Pantoea agglomerans* are usually associated with an identifiable exogenous source [7]. These organisms grow well at 4 °C and most commonly cause septic arthritis or synovitis following a penetrating injury by vegetation. Organic materials like plant thorn may penetrate the skin and remain embedded in the tissues and set up a chronic inflammatory process [8, 9].

Although *Escherichia coli* is a common cause of vertically transmitted infection in the new born, *Pantoea agglomerans* infection could also be the result of exposure to colonizing bacteria in the birth canal following PROM. A similar case was reported by Lalas and Erichsen in 2010 [6]. Although it is very difficult to draw any conclusion from a single case, it should be kept in mind that *Pantoea* spp*.* could be a rare cause of vertically transmitted infection in a term baby.

## Conclusions

*Pantoea* spp*.* is a rare pathogen in the etiology of early onset neonatal sepsis. In the majority of previous cases, the outcome was very poor because of association with prematurity and comorbidity. In our case the outcome was excellent because it was a term baby, the antibiotic meropenem was administered intravenously and other supportive measures like oxygen were given in time. Early detection and proper antibiotic therapy may cause a favorable outcome despite significant clinical deterioration.

## Consent

Written informed consent was obtained from the patient’s legal guardian(s) for publication of this case report and any accompanying images. A copy of the written consent is available for review by the Editor-in-Chief of this journal.

## Abbreviations

NICU: Neonatal intensive care unit
PROM: Premature rupture of membrane
Spp.: Species
